# Psychoactive Substances of Natural Origin: Toxicological Aspects, Therapeutic Properties and Analysis in Biological Samples

**DOI:** 10.3390/molecules26051397

**Published:** 2021-03-05

**Authors:** Joana Gonçalves, Ângelo Luís, Eugenia Gallardo, Ana Paula Duarte

**Affiliations:** 1 Centro de Investigação em Ciências da Saúde (CICS-UBI), Universidade da Beira Interior, Av. Infante D. Henrique, 6200-506 Covilhã, Portugal; joanadgoncalves13@gmail.com; 2 Laboratório de Fármaco-Toxicologia, UBIMedical, Universidade da Beira Interior, Estrada Municipal 506, 6200-284 Covilhã, Portugal

**Keywords:** NPS of natural origin, psychoactive effects, toxicological aspects, traditional uses, analytical methodologies

## Abstract

The consumption of new psychoactive substances (NPSs) has been increasing, and this problem affects several countries worldwide. There is a class of NPSs of natural origin, consisting of plants and fungi, which have a wide range of alkaloids, responsible for causing relaxing, stimulating or hallucinogenic effects. The consumption of some of these substances is prompted by religious beliefs and cultural reasons, making the legislation very variable or even ambiguous. However, the abusive consumption of these substances can present an enormous risk to the health of the individuals, since their metabolism and effects are not yet fully known. Additionally, NPSs are widely spread over the internet, and their appearance is very fast, which requires the development of sophisticated analytical methodologies, capable of detecting these compounds. Thus, the objective of this work is to review the toxicological aspects, traditional use/therapeutic potential and the analytical methods developed in biological matrices in twelve plant specimens (*Areca catechu*, *Argyreia nervosa*, *Ayahuasca*, *Catha edulis*, *Datura stramonium*, *Lophophora williamsii*, *Mandragora officinarum*, *Mitragyna speciosa*, *Piper methysticum Forst*, *Psilocybe*, *Salvia divinorum* and *Tabernanthe iboga*).

## 1. Introduction

The use of drugs of abuse is a concern that has been increasing over the years. About 96 million individuals have already used drugs of abuse in the European Union, cannabis being the most used (27.4%), followed by cocaine (5.4%) and then ecstasy (4.1%) and amphetamines (3.7%) [1]. In recent years, a trend of new psychoactive substance (NPS) consumption has been reported. The European Monitoring Center for Drugs and Drug Addiction (EMCDDA) defines those compounds as “a new narcotic or psychotropic drug, in pure form or in preparation, that is not controlled by the United Nations Drug Conventions, but which may pose a public health threat comparable to that posed by substances listed in these conventions” [2]. NPSs have spread around the world, mainly because they are marketed on the internet in dark web forums, with different names, namely “bath salts”, “legal highs”, or “research chemicals” [3]. These substances are generally consumed because they are able to mimic the effects of more conventional drugs of abuse and because they are not detected in common screening methods [4,5,6]. However, the various risks associated with the consumption of NPSs are described in the literature, as well as the resulting health problems [7,8,9].

Despite the term “new” referring to a recent appearance, the truth is that some of these compounds have existed for decades, but they only became available on the market more recently, and consequently, their commercialization is not yet regulated [3]. The constant appearance of these drugs (about 50 new per year) is a concern in terms of controlling their marketing [1]. Additionally, considering the health hazard presented by NPSs, the United Nations Office on Drugs and Crime (UNODC) and the EMCDDA have implemented early warning systems in order to detect these compounds [10,11].

NPSs may have a synthetic or natural origin, the most recognized synthetic NPS being synthetic cannabinoids, cathinones and opioids, piperazines, phenethylamines, designer benzodiazepines, indoalkylamines and arylcyclohexylamines [2,3,4]. NPSs of natural origin consist mainly of alkaloids naturally present in plants that, when consumed, allow the user to experience new sensations and different “mental states” [3,12]. These plants come mainly from South America and Asia but also from Africa and Russia [3] and, depending on their constituents, can trigger relaxing and/or sedative effects, such as *Areca catechu* (*A. catechu*) and *Mitragyna speciosa* (*M. speciosa*), hallucinogenic effects, as the constituents of *Ayahuasca*, or stimulating effects, such as *Catha edulis* (*C. edulis*) [3,13]. The consumption of preparations containing alkaloids of natural origin is often prompted by religious beliefs or cultural reasons, making it difficult to estimate the worldwide consumption of these substances [3]. For these reasons, the legislation that regulates these substances is quite variable and may even be ambiguous [2].

The metabolism of these substances is not fully studied, and therefore the resulting metabolites and their potential concentrations are not known [2,3]. Another gap that needs further study is the acute toxicity of many of these substances, which are also not completely known [2]. In fact, the symptoms described during intoxication with an NPS are confused with symptoms of consumption of other substances, namely medicines [2]. Thus, developing analytical methodologies is greatly important for the detection and quantification of potentially dangerous compounds present in these natural products. However, most developed analytical methods have focused on the detection of alkaloids naturally present in plant materials [14].

In this review, we sought to address the toxicological aspects of several psychoactive substances present in different plants, as well as some therapeutic properties/traditional uses. In addition, the analytical methods developed in biological samples aimed at the detection of psychoactive substances from the same plants were also discussed.

## 2. *Areca catechu* (Betel Quid)

**Figure 1:**
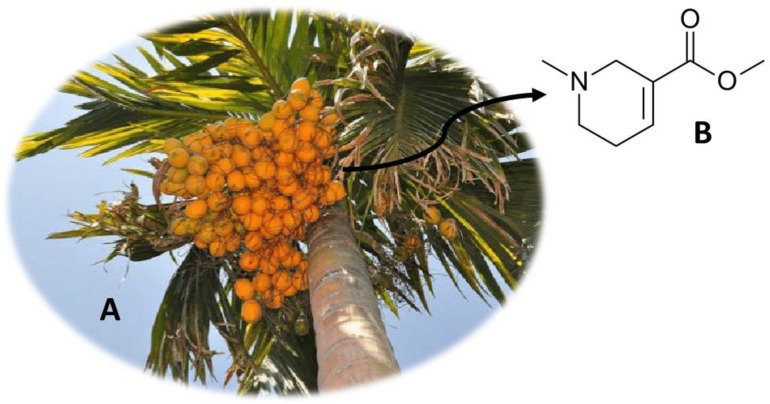
*A. catechu* (**A**) and the main compound arecoline (**B**).

Currently, *A. catechu* (Figure 1A) is distributed in Africa, Europe and America, in spite of its main origin being Asia (Sri Lanka and Malaysia) [2]. The areca nut is the fruit produced by this plant, having been consumed for centuries as a traditional remedy or in rituals [15]. This fruit is normally chewed and can be consumed together with other substances in the form of a “betel quid” [15,16]. Areca nut is the fourth drug with the highest consumption rate worldwide, possibly due to its stimulating, relaxing or aphrodisiac effects [15,16]. Arecoline (Figure 1B) is the main psychoactive compound present in the fruit of *A. catechu* [2]. This compound is an alkaloid that works as a competitive inhibitor of gamma-aminobutyric acid (GABA) and as a non-selective nicotinic and muscarinic agonist [3,17,18]. Once in the body, arecoline quickly crosses the blood-brain barrier, exerting effects on the parasympathetic nervous system [3]. However, this fruit is addictive and can cause several adverse effects, namely on the digestive system and abstinence syndrome (insomnia, mood swings, irritability and anxiety) [3,15]. Other effects, such as severe extrapyramidal syndrome, asthma and myocardial infarction, have also been associated with the consumption of this fruit [19,20]. However, the use of this fruit for medicinal purposes has also been described, since antiquity, by Hindu and Buddhist peoples [21]. The consumption of areca nut has been associated with general properties such as satisfaction, well-being, psychostimulating effects, stress reduction, gum strengthening and breath sweetening [21]. Additionally, this fruit is used in the treatment of malaria, fever, hernia, hypertension, urinary stones and in the manufacture of formulations for the treatment of digestive diseases, diarrhea and indigestion [21]. Studies have also indicated that the consumption of areca nut is associated with antimicrobial [22,23], cardiovascular [23,24] and digestive effects [21,23,24,25]. In addition to the *A. catechu* fruit, roots and leaves were also traditionally used in medicine [21].

*A. catechu* and its fruit are not controlled substances, and therefore there is no legislation for their consumption in the United States of America and in the European Union [3]. Thus, several analytical methods, which allow the measurement of these substances, have been developed (Table 1). Conventional samples, such as blood, continue to be used for the detection of the most diverse compounds, including arecoline [26]. Wu et al. [26] developed an analytical method where they proceeded to quantify arecoline in an LC-MS/MS equipment, using 1 mL of blood, obtaining a limit of detection (LOD) of 0.02 ng/mL and a limit of quantification (LOQ) of 0.5 ng/mL. Urine is another biological matrix that has been used for the measurement of arecoline [27]. Pichini et al. [27] developed an analytical method in HPLC-MS equipment, where they used 1 mL of urine, having managed to quantify arecoline. In the same study, it was also possible to quantify the same compound in samples of meconium (1 g) and cord serum (1 mL) [27]. However, other alternative samples, such as teeth [28], saliva [29,30] and breast milk [31] have been also used in the development of new analytical methods for detecting arecoline. Pellegrini et al. [31] developed a method for the determination of arecoline in LC-MS/MS, using 1 mL of breast milk. This method had a LOD of 16 ng/mg and LOQ of 50 ng/mg [31].

## 3. *Argyreia nervosa* (Adhoguda)

**Figure 2:**
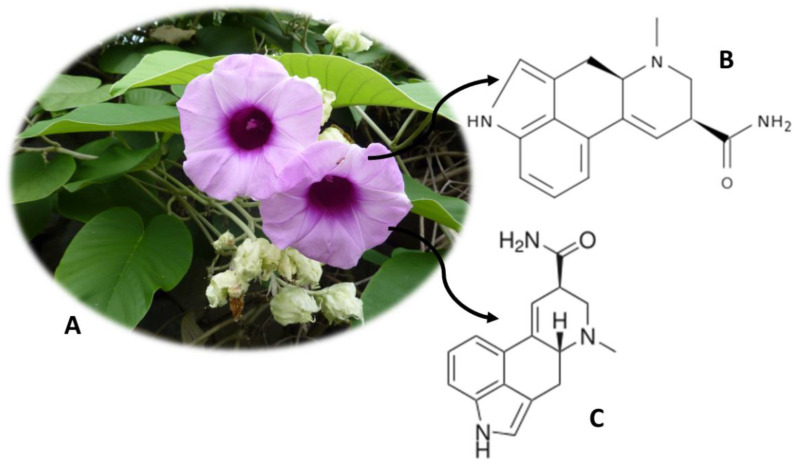
*Argyreia nervosa* (**A**) and the main compounds isoergine (**B**) and lysergamide (LSA) (**C**).

*A. nervosa* (Figure 2A) is originally from India, but it is widely distributed in Europe, Africa and subtropical America [33]. This plant, also known as Adhoguda, Vidhara, Elephant Creeper, *Rivea corymbosa*, Hawaiian Baby Woodrose or Morning Glory or *Ipomoea violacea*, possesses psychoactive alkaloids in its seeds [3]. Isoergine (Figure 2B) and lysergamide (LSA) (Figure 2C) are the compounds responsible for the hallucinogenic properties of this plant, being able to induce effects similar to lysergic acid diethylamide (LSD), albeit with a lower intensity [34]. *A. nervosa* seeds have a total of ergoline alkaloids between 0.5% and 0.9%, of which 0.19% correspond to isoergine and 0.14% correspond to LSA [35]. The LSA exerts its effects by binding to dopamine D2 receptors and consequent inhibition of adenylate cyclase and reduction in the production of cyclic adenosine monophosphate (cAMP) [36]. The consumption of this plant for medicinal purposes has also been described, namely as a diuretic and aphrodisiac [13]. Analgesic, anti-inflammatory, immunomodulatory, hepatoprotective and hypoglycemic properties have also been described [13,37]. *A. nervosa* roots are also used in the treatment of diseases of the central nervous system, rheumatism, gonorrhea and chronic ulcer. On the other hand, antimicrobial activity has been associated with the plant leaf [13,37]. In addition to the two alkaloids already mentioned, others have been also isolated from plants, namely erginine, ergometrine, lysergol, peniclavine, chanoclavin I, chanoclavin II, ergometrinine, elimoclavin and egine, but their effects are not yet known [38].

LSA is a controlled substance in some European countries, namely in the United Kingdom and Italy. It is also controlled in the United States of America, but the plant and its seeds are freely sold [3]. There are currently analytical methodologies developed that allow quantifying the LSA. Paulke et al. [39] developed an analytical method, with 1 mL of serum and urine, to detect and quantify LSA. The analytes were extracted using the SPE (solid-phase extraction) technique and quantified on HPLC-FLD equipment [39]. The method had detection and quantification limits of 0.05–0.15 ng/mL and 0.17 ng/mL, respectively, and recoveries between 69.4% and 78.8% [39].

## 4. Ayahuasca (“Hoasca”)

**Figure 3:**
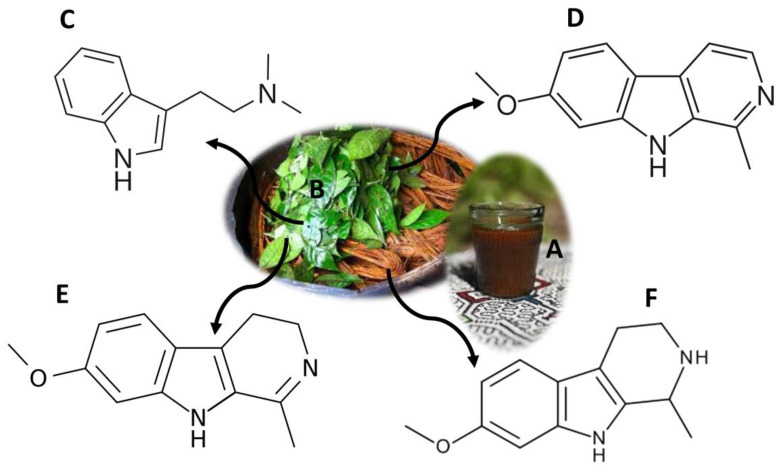
Ayahuasca decoction (**A**); *Banisteriopsis caapi* stem shavings and *Psychotria viridis* leaves used in the preparation of the Ayahuasca beverage (**B**); main compounds present in Ayahuasca: DMT (**C**), Harmine (**D**), Harmaline (**E**) and THH (**F**).

Ayahuasca is a word of Quechua origin, composed of two terms: “aya” and “waska”, which mean “spirit” and “vine”, respectively [40]. On its whole, the word Ayahuasca means “rope of the soul”, and it is also known as caapi, daime, hoasca, yagé and natema [3,40]. This term refers to a psychoactive drink, traditional in South America (Figure 3A). More recently, it has been imported into some countries in Europe and Asia [3,41,42]. Ayahuasca consists of a brown, thick and oily liquid, the result of a decoction of shavings from the stem of *Banisteriopsis caapi* (*B. caapi*) and leaves of *Psychotria viridis* (*P. viridis*) (Figure 3B) [41,42]. Additionally, other species of natural origin, which replace those already mentioned, can be used in the preparation of Ayahuasca, namely *Brugmansia suaveolens*, *Psychotria carthagenensis*, *Nicotiana tabacum*, *Tabernaemontana* spp., *Brunfelsia* spp., *Datura suaveolens*, *Iochroma fuchsioides*, *Malouetia tamarquina*, *Juanulloa* spp. and *Peganum harmala*, among other products with hallucinogenic compounds [43].

The effects of this psychoactive mixture are due to the synergy potential of N,N-dimethyltryptamine (DMT) (Figure 3C), a hallucinogenic compound from *P. viridis*, and of the harmine (Figure 3D), harmaline (Figure 3E) and tetrahydroharmine (THH) (Figure 3F), which are *β*-carbolinic alkaloids present in *B. caapi* [44,45]. DMT is a tryptamine that acts as an agonist for serotonin receptors (5-HT1A/2A/2C) [3]. When this compound is ingested alone, it undergoes metabolism by peripheral monoamine oxidase A (MAO-A), being inactive [46]. However, when DMT is ingested together with *β*-carbolinic alkaloids, it is able to penetrate the central nervous system, since it temporarily inhibits MAO-A [44,46,47,48]. In addition, THH also inhibits serotonin reuptake by increasing the effects of DMT [49]. Users describe visual hallucinations, with effects on temperature, pupil size and changes in the endocrine, cardiovascular and immune systems [3,50]. Side effects such as mydriasis, vomiting, hypertension, tachycardia, agitation, paranoia, anxiety and depression have also been described [3,50,51]. However, there are several studies that report therapeutic properties. Recently their proprieties were reported as antimicrobial and antioxidant agents [52], as well as their effect over dopaminergic neuron cells [53]. Studies have shown that a single dose of Ayahuasca leads to a rapid reduction in depressive symptoms, and this reduction is maintained for three weeks [54,55]. Other studies show that the consumption of this decoction results in a significant reduction in anxiety and panic [54,56]. The reduction of drug and alcohol abuse [57,58,59], attention problems [60] and decreased physical pain, fatigue, insomnia, irritability and obsession [61] have also been described.

Ayahuasca has been used in religious rituals in the Amazon for centuries, and more recently by religious entities such as União do Vegetal (UDV) and Santo Daime [3,40]. DMT-containing substances are controlled in the United States of America and in some European countries [3]. However, the consumption of *P. viridis* and *B. caapi* is not controlled, and the use of Ayahuasca for religious purposes is legal in the United States of America and Brazil [3]. There are currently several analytical methodologies that allow the detection and quantification of the compounds from Ayahuasca and its metabolites (Table 2). The samples of choice for the quantification of these compounds are the so-called conventional samples, namely blood [62], plasma [63,64] and urine [65,66]. Yritia et al. [63] and Oliveira et al. [64] developed analytical methods for the detection of DMT and *β*-carbolines, using 1 mL of plasma. Both methods used SPE [63,64] as a sample pre-treatment technique, and in the first study, a liquid-liquid extraction (LLE) was also performed [63]. Both studies showed good limits of detection and quantification, as well as good recoveries [63,64]. More recently, Pichini et al. [67] carried out a study, where they quantified DMT, using only 25 mg of hair. The hair sample was initially hydrolyzed with an M3 reagent, and HPLC-MS-MS equipment was used to quantify the analyte [67]. The LOD varied between 0.01 ng/mg and 0.02 ng/mg and the LOQ between 0.03 ng/mg and 0.05 ng/mg, with recoveries between 76.6% and 97.4% [67].

## 5. *Catha edulis* (Khat)

**Figure 4:**
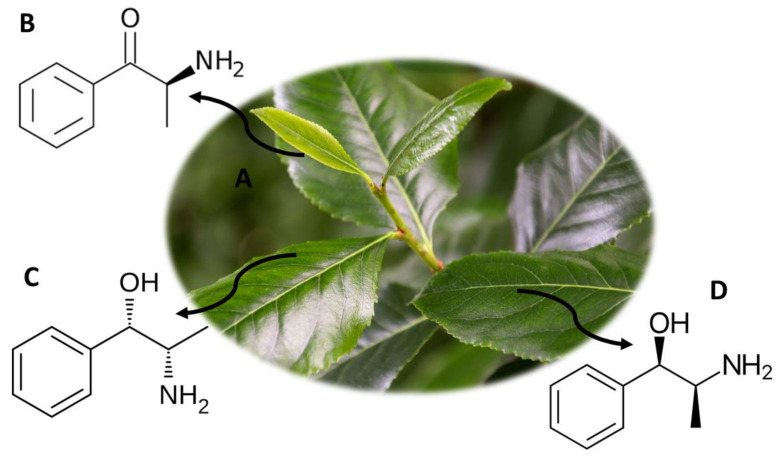
*C. edulis* (**A**) and the main compounds S-(−)-cathinone (**B**), cathine ([S,S-(+)-norpseudoephedrine]) (**C**) and phenylpropanolamine (**D**).

*C. edulis* (Figure 4A) comes from some West African countries, as well as from Yemen, Ethiopia and the Arabian Peninsula [2]. This plant is often used as a drug of abuse since it allows to mimic the effects of synthetic cathinones but with a lower risk of intoxication, with no record of deaths associated with its consumption [2]. *C. edulis* is also called khat, qat and kafta, among others, and it is usually consumed in smoked form or by chewing fresh leaves [3]. The psychoactive components present in the leaves of this plant are S-(−)-cathinone (Figure 4B), cathine ([S,S-(+)-norpseudoephedrine]) (Figure 4C) and phenylpropanolamine (Figure 4D). S-(−)-cathinone is photosensitive, and therefore it degrades easily with sun exposure, being the major compound in fresh khat leaves, but it is not found in older leaves [68]. After sun exposure, S-(−)-cathinone degrades into chatine and (−)-norephedrine, these being the compounds present mostly in the older leaves [68]. When consuming khat, the active compounds degrade not only into chatine and (−)-norephedrine but also into [R,S-(−)-norephedrine] and [R,R-(−)-norpseudoephedrine], compounds structurally similar to amphetamine [69]. Consumers of this plant describe effects such as hyperthermia, euphoria, increased breathing and sensory stimulation, excitation and anorexia. However, adverse effects such as violent behavior, schizophrenia, paranoia and psychosis, increased blood pressure, insomnia, tachycardia, irritability, migraine and sexual dysfunction have also been described [3].

The consumption and trade of khat leaves are not regulated by any international system, but the consumption and trade of cathinone and cathine are prohibited worldwide [3]. In some countries, khat is considered a controlled substance, namely Ireland, France, Germany, Denmark and the United States of America. In the Netherlands, its trade is not prohibited, but is restricted and, in Canada, the possession of khat is allowed, but its import and trade are also illegal [3]. On the other hand, countries like Yemen, Somalia and Ethiopia allow the consumption of khat, since it is a cultural habit [3].

There are currently analytical methodologies developed that allow quantifying the compounds present in khat (Table 3). Sørensen [70] developed an analytical method to quantify 15 compounds, using LC-MS/MS equipment with only 300 µL of blood. The sample was treated with methanol to precipitate proteins, and then it was filtered [70]. The analytical method showed recoveries between 87% and 106% and a LOD between 0.5 ng/mL and 3 ng/mL [70]. In addition, samples such as plasma [71], urine [72] and oral fluid [73] were used to quantify these compounds. Mohamed et al. [73] used 500 µL of oral fluid to quantify cathine, methcathinone, cathinone and ephedrine. The samples were submitted to an LLE (ethyl acetate) extraction technique and subsequently analyzed by GC-MS. The analytical method showed a LOQ of 20 ng/mL and a LOD of 10 ng/mL [73].

## 6. *Datura stramonium* (Jimson Weed)

**Figure 5:**
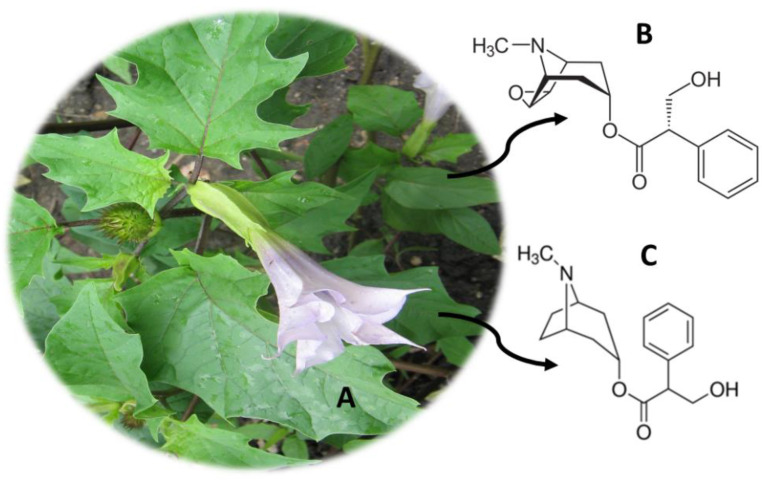
*Datura stramonium* (**A**) and the main compounds scopolamine (**B**) and atropine (**C**).

*D. stramonium* (Figure 5A), originally from the United States of America, consists of a seasonal herb that grows naturally [2]. This species, also known as Jimson Weed, was traditionally used by the Pueblo Indians, due to its analgesic properties [76]. Moreover, in Western medicine, Jimson Weed was used to treat asthma [76]. This plant is usually consumed by eating its seeds or flowers intact, or in the form of an infusion of leaves or crushed seeds [77,78]. Dried leaves, flowers and seeds are also consumed in a smoked form, and there is also Asthmador™ powder available for consumption in smoked form or by inhalation [78,79]. The consumption of *D. stramonium* also causes hallucinogenic effects, which are due to the presence of the alkaloids scopolamine (Figure 5B) and atropine (Figure 5C) distributed throughout the plant [2]. These compounds are tertiary amines and therefore cross the blood-brain barrier rapidly [79]. Scopolamine acts at the level of the central nervous system, exerting antimuscarinic effects [80]. The effects of Jimson Weed consumption include tachypnea, delirium, psychomotor agitation, dilation of the pupils, blurred vision or photophobia [78,79,81]. Other effects, such as peripheral vasodilation, decreased thermoregulation, vomiting, constipation and difficulties in urinating, have been also described [76,82]. At higher doses, respiratory depression and even cardiac arrest, seizures or hypoventilation may occur [79]. Some analytical methods have been developed to quantify the alkaloids scopolamine and atropine (Table 4). These are discussed below.

## 7. *Mandragora officinarum* (Mandrake)

**Figure 6:**
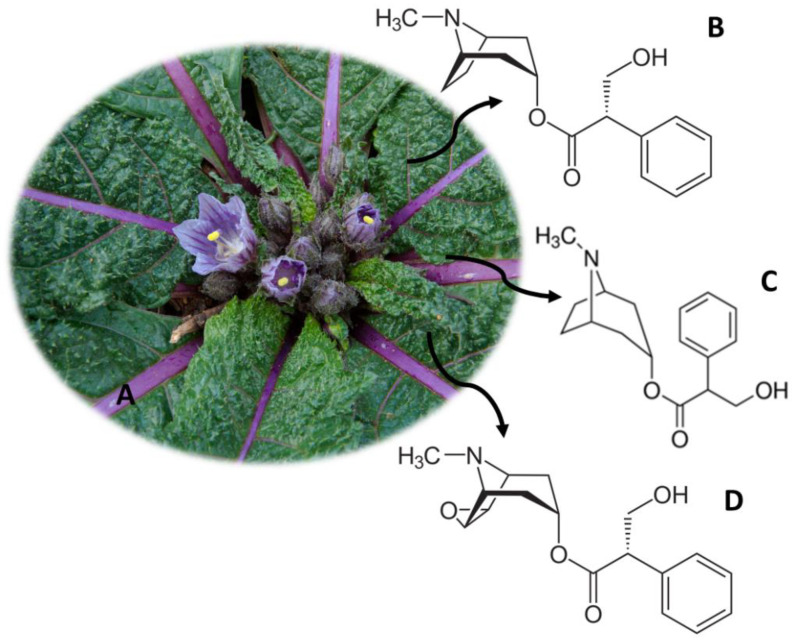
*M. officinarum* (**A**) and the main compounds hyoscyamine (**B**), atropine (**C**) and scopolamine (**D**).

*M. officinarum* (Figure 6A) is widely distributed worldwide, namely in Europe, North Africa, the Middle East and the Himalayas, however originating from the eastern Mediterranean [2]. This plant, also known as mandrake, has in the constitution of its seeds, roots, leaves and fruits, hyoscyamine (Figure 6B), atropine (Figure 6C) and scopolamine (Figure 6D), responsible for its healing, hallucinogenic and poisonous properties [83,84]. Since ancient times, mandrake was used as a surgical anesthetic in Rome and Greece [85]. It is also believed that this plant has aphrodisiac properties and its fruit increases fertility [85].

The consumption of mandrake can compromise the autonomic nervous system, resulting in an anticholinergic action and, consequently, reducing neuronal activity mediated by acetylcholine [86,87]. Thus, effects such as dry mouth, urinary retention, increased heart rate, mydriasis and decreased secretions are described [3,86,87]. In more extreme cases, its consumption can induce coma or even cause death [3]. Thus, the use of this plant is controlled both in the United States of America and in Europe, with the imposed measures being very restrictive [3].

There are currently analytical methodologies developed with different biological samples, which allow the quantification of atropine and scopolamine (Table 4). Pietsch et al. [88] developed an analytical method with 1 mL of serum and urine to detect and quantify 13 compounds, namely scopolamine and atropine. The analytes were extracted using the SPE technique and quantified using HPLC-PDA and HPLC-UV equipment [88]. The method presented quantification limits of 0.3–94 ng/mL and recoveries between 23.7% and 86.9% [88]. In addition, Carlier et al. [89] quantified atropine and scopolamine, among other compounds, in a single analytical method. The SPE technique was used as a pre-treatment of the blood sample (1 mL), having subsequently been quantified in UHPLC-MS/MS equipment [89]. The method had a LOQ of 10 ng/mL and detection limits between 0.1 and 1.6 ng/mL [89].

## 8. *Lophophora williamsii* (Peyote)

**Figure 7:**
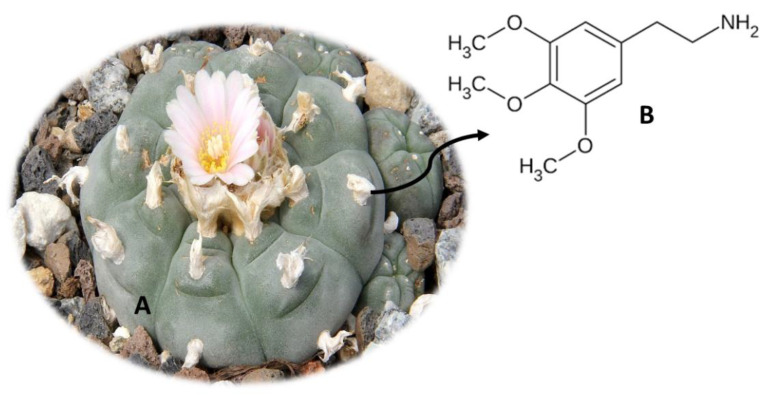
*Lophophora williamsii* (**A**) and the main compound mescaline (**B**).

*L. williamsii* (Figure 7A), also known as Peyote, is a cactus from northern Mexico and the United States of America [92,93]. This plant was traditionally eaten in religious rituals, by indigenous peoples in the countries already mentioned [50]. Normally, the flesh of the fresh cactus is ingested, and it can also be dried and subsequently ingested or used to make teas [92,94]. *L. williamsii* contains a compound called mescaline [2-(3,4,5-trimethylphenyl) ethanamine] (Figure 7B), a member of the phenylalkylamine class, responsible for the hallucinogenic properties of the plant [79,95]. This compound is also found for sale in the form of powder, which can be inflated or ingested orally [79]. Once consumed, mescaline accesses the central nervous system, acting at the level of serotonergic receptors 5-HT2 as an agonist of subtypes 5-HT2a, 5-HT2b and 5-HT2c [95].

The effects when consuming this plant include paranoia, compulsion, paresthesia, changes in color perception, headaches, mydriasis, spasms and psychomotor agitation [93,96,97]. Other effects at the cardiovascular, gastrointestinal and renal levels have been also described, namely hypertension and tachycardia, vomiting and decreased filtration rate at the glomerular level [93,98,99]. However, beneficial effects have been also described. One study demonstrated that *L. williamsii* extracts were effective in treating rheumatism, wounds, burns and snakebites [100]. Another study showed that this plant has antimicrobial properties against *Staphylococcus aureus* [100] Additionally, *L. williamsii* is used by some tribes to treat fever, labor pain, toothache, diabetes, blindness, breast pain and skin diseases [100].

Currently, substances containing mescaline are included in Annex I of the 1967 United Nations Convention on Drugs [100]. Given the effects of this plant, it is crucial to develop new analytical methodologies to detect mescaline and its metabolites in biological samples. Until now, methodologies have been developed using chromatography, namely in alternative samples such as hair [67]. Pichini et al. [67] developed an analytical method, using UHPLC-MS/MS equipment, to quantify several naturally occurring hallucinogens, including mescaline. For this purpose, 25 mg of hair was hydrolyzed with an M3 reagent. The method presented LOD values between 0.01 ng/mg and 0.02 ng/mg, LOQ between 0.03 ng/mg and 0.05 ng/mg and recoveries between 79.6% and 97.4% [67]. Another study by Beyer et al. [71] also allowed to quantify mescaline, using LC-MS/MS equipment with 1 mL of plasma, pre-treated with the SPE technique.

## 9. *Mitragyna speciosa* (Kratom)

**Figure 8:**
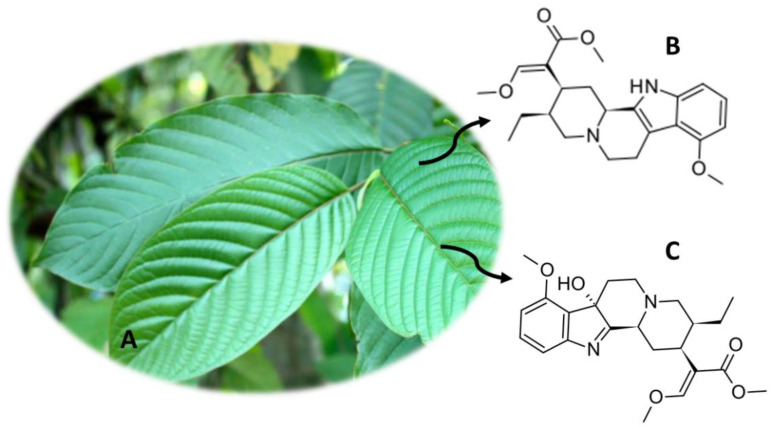
*M. speciosa* (**A**) and the main compounds mitragynine (**B**) and 7-hydroxymitragynine (**C**).

*M. speciosa* (Figure 8A), also known as Kratom, appeared on the Asian continent, namely in countries like Biak, Malaysia and Thailand [2]. Currently, this plant is distributed in several regions of the world [3,12]. *M. speciosa* has been used for several years by rural workers and peasants in Asian people for reducing fatigue and increasing productivity at work, coughing, pain, fever, diarrhea, hypertension and diabetes. More recently, it began to be consumed in a recreational context in Europe and the United States of America [101,102,103,104,105,106,107,108,109]. Kratom leaves have been also used as a substitute for opium, as well as in morphine withdrawal treatments [12,50]. The preferred mode of consumption is chewing fresh leaves, but dried leaves can also be eaten or smoked [3,50]. Other forms of consumption of this plant include the preparation of teas and pastes by boiling the leaves for a long period [16]. Currently, there is greater ease in the consumption of this plant, since capsules, powders and drinks are available that can be easily purchased [3,110].

Kratom has psychoactive properties, which are due to the presence of about 40 alkaloids in the plant [3,50]. These compounds correspond to about 0.5%–1.5% of the compounds and their concentrations vary with the harvesting season, age and geographic location [3,111]. The most abundant psychoactive compound is mitragynine (Figure 8B), corresponding to a total of 66.2% of the alkaloids content. However, the abundance of this compound in Malaysian plants was only 10% [12]. Other alkaloids with pharmacological activity were also detected, such as 7-hydroxmitraginine (Figure 8C) and corinantheidine [110,111,112,113,114]. In addition, other alkaloids have been discovered that may contribute to pharmacological effects, namely corinantheidine, specioginine and paynantheine [3]. The alkaloids present in *M. speciosa* show high lipophilicity, crossing the blood-brain barrier, and a high affinity for opioid receptors [3]. Thus, 7-hydroxmitraginin binds to the supraspinous k-opioid and µ-opioid receptors, exerting their effects [3]. In addition to these, mitragynine binds to δ-opioid receptors, thereby exercising analgesic effects [3,50]. Mitragynine is able to block Ca^2+^ channels, affecting the release of neurotransmitters [115,116]. Thus, antidepressant, antioxidant and anti-inflammatory properties have been associated with kratom consumption [117,118]. The use of this substance for substitution treatment in chronic opioid users has also been reported [3,50].

Adverse effects when consuming this plant include withdrawal and neonatal withdrawal syndrome, seizures, weight loss, dehydration, fatigue, insomnia, constipation and hyperpigmentation [50,103,105,119,120]. However, *M. speciosa* is not on the United Nations Drug Convention Schedule [3]. These compounds are controlled in the United States of America, New Zealand, Australia, Myanmar, Thailand, Malaysia and in some European countries [3].

Thus, it is crucial to develop analytical methods to detect and quantify the compounds present in *M. speciosa* (Table 5). Carlier et al. [89] developed an analytical method where they used 1 mL of blood to detect mitragynine (among other compounds), using UHPLC-MS/MS equipment. Lee et al. [121] developed an analytical method in LC-MS/MS, where they used SPE and enzymatic hydrolysis as a method of pre-treatment of the urine sample, to quantify 16-carboxy mitragynine, 9-*O*-demethyl mitragynine and mitragynine. More recently, Basiliere et al. [122] developed an analytical method using LC-Q/TOF-MS equipment for the quantification of mitragynine, 7-hydroxymitragynine, among other compounds. One milliliter of urine, pre-treated with SPE, was used, obtaining a LOD of 0.25–1 ng/mL and a LOQ of 0.5–1 ng/mL [122].

## 10. *Piper methysticum* Forst (Kava)

**Figure 9:**
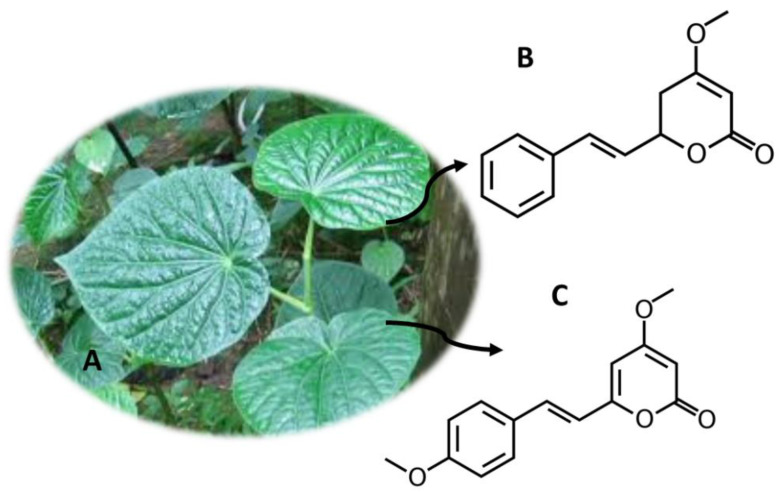
*P. methysticum* (**A**) and the main compounds kavain (**B**) and yangonin (**C**).

Some parts of *P. methtysticum* (Figure 9A) (roots and stems) are used in the manufacture of Kava, a psychotropic drink from the Pacific region [2]. Kava was consumed due to its therapeutic properties, namely in reducing fatigue and anxiety, relieving pain or inducing sleep [125]. Other treatments such as restlessness and anxiety were also associated with the consumption of Kava [3]. However, the use of this substance is associated with hepatotoxicity [125]. The effects of Kava are due to kavalactones, namely kavain (Figure 9B), yangonin (Figure 9C), desmethoxy-iangonin, 7,8-dihydrokavain, methysticin and 7,8-dihydromethysticin, to the derivatives of cinnamic acid, flavanones and chalcones [2]. These compounds act at the level of the central nervous system, inhibiting monoamine oxidase B, recapturing of noradrenaline and dopamine and interacting with γ-amino butyric acid [126].

The sale of *P. methysticum* is controlled in Holland, Switzerland and France, and its sale and import are prohibited in the United Kingdom. In Poland, sales for human consumption are also prohibited. However, in most countries, this substance remains legal [3]. Thus, the development of analytical methods for the detection of these compounds is becoming increasingly important (Table 6). Villain et al. [127] developed a method for the determination of kavain in GC-MS/MS, using between 29 and 50 mg of hair. The method had a LOD of 30 ng/g and a LOQ of 100 ng/g [127]. Another more recent study, carried out by Tarbah et al. [128], allowed to quantify 10 compounds, using between 21 and 253 mg of hair. The sample was initially decontaminated, then digested, using three different types of equipment for quantification: HPLC-DAD, LC-MS/MS and GC/TOF-MS [128].

## 11. *Psilocybe* Genus (Magic Mushrooms)

**Figure 10:**
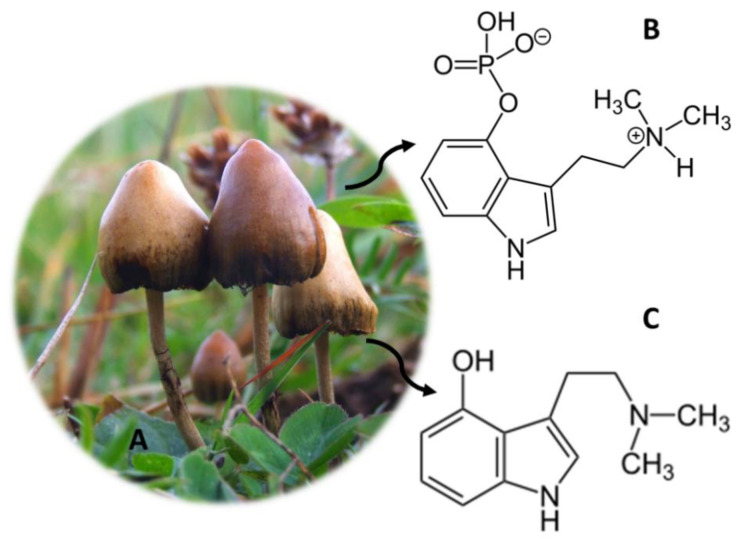
*Psilocybe* mushrooms (**A**) and the main compounds psilocybin (**B**) and psilocin (**C**).

*Psilocybe* (Figure 10A) is a genus of hallucinogenic fungi, commonly known as magic mushrooms [131]. These specimens originate from certain regions of South America, but it is also possible to find them in Western Europe and in the United States of America [79,92]. The magic mushrooms were initially used in religious rituals, by the Aztec people in Mexico, persisting until today [50,79]. The active compounds present in these fungi are psilocybin (Figure 10B) and psilocin (Figure 10C), which consist of a substituted indolealkylamine [3,79]. There are about 190 species of mushrooms of the genus *Psilocybe*, which contain these two compounds responsible for the psychoactive effects of these fungi [131]. Mushrooms can be eaten after drying and making tea, but the most common route of consumption is by eating whole mushroom capsules [92]. After being consumed, psilocybin is converted into psilocin, and it acts as an agonist for the serotonergic receptors 5-HT1a and 5-HT2a, exerting its psychoactive effects [79]. In addition, these compounds can also increase the release of glutamate, which activates receptors such as *N*-methyl-d-aspartic acid receptors and α-amino-3-hydroxy-5-methyl-4-isoxazolepropionic acid receptors [50,132]. The effects caused when consuming magic mushrooms can also be partially and indirectly mediated by dopamine [133].

The effects of *Psilocybe* consumption include changes in perception similar to drugs such as LSD, namely changes in visual and auditory perception [3]. Mystical experiences, tachycardia, headache, sweating, mydriasis, chills, nausea and increased body temperature are also associated with the consumption of magic mushrooms [134,135]. Other effects reported when consuming these substances are paranoia, dizziness and imbalance and abdominal pain, [79,135]. Moreover, it has been reported that psilocybin can be used to treat anxiety and resistant depression [136].

*Psilocybe* mushrooms are illegal all over the world [137]. However, in some countries, the law is not consensual, namely in the Netherlands where the mushroom is illegal, but the Sclerotia truffle (philosopher’s stone) is not [3]. Given the worldwide consumption of this substance, the development of new analytical methods that allow the determination of these compounds is crucial (Table 7). Several samples were used to quantify the active compounds of *Psilocybe*, namely urine [91,138], serum [139] and hair [67]. Kamata et al. [139] developed a method for the quantification of psilocin glucuronide, where they used only 100 µL of serum. The sample was subjected to an enzymatic hydrolysis and deproteinization process, after which it was injected into LC-MS and LC-MS/MS equipment. The method showed 0.5 ng/mL LOD. The same authors had previously developed an analytical method for the quantification of psilocin glucuronide and psilocin, where they used the same volume of urine and the same equipment, and the same LOD was obtained [138].

## 12. *Salvia divinorum* (“Hierba de Maria”)

**Figure 11:**
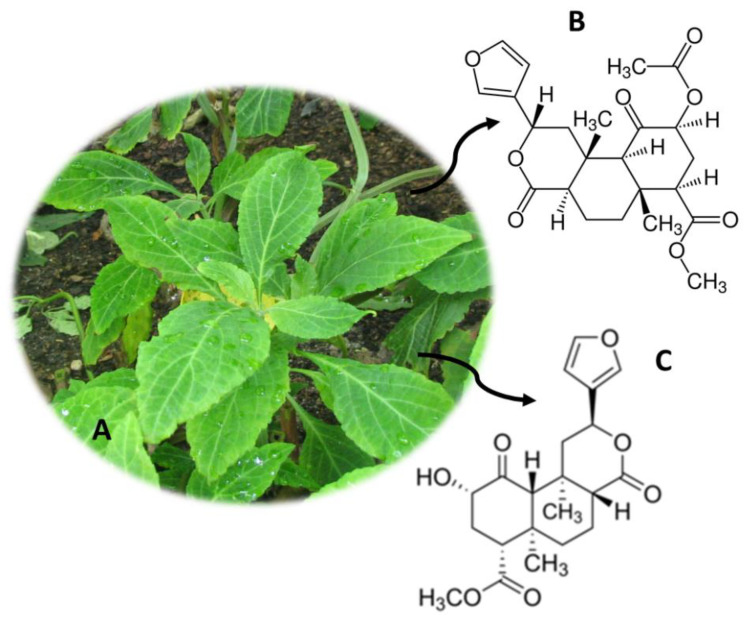
*S. divinorum* (**A**) and the main compounds salvinorin A (**B**) and salvinorin B (**C**).

*S. divinorum* (Figure 11A) originates from Oaxaca, a region in the northeast of the Sierra Mazateca, Mexico [140]. This psychoactive plant, also known as hierba de Maria, hojas de la Pastora, ska Maria, ska Pastora and magic mint, has been used for centuries by indigenous people because they believe it is the reincarnation of the Virgin Mary [12,141]. *S. divinorum* is consumed by chewing fresh or dried leaves. Dried leaves can be also smoked, and fresh leaves can be used to make tea [3]. The main psychoactive constituent of this plant is salvinorin A (Figure 11B), but other compounds were also detected, such as salvinorins B (Figure 11C), C, D, E and F, but these do not have pharmacological activity [3,12]. Salvinorin A acts as a selective agonist for Kappa opioid receptors (KOR), thereby exerting its potent hallucinogenic effects [12,142]. A dose of between 200 μg and 500 μg is capable of inducing deep hallucinations with extraordinary illusions and a feeling of physical and mental displacement [143,144]. However, some studies conducted with *S. divinorum* and its bioactive compound salvinorin A have shown that it has some effects with therapeutic potential, such as drug addiction, pain treatment, neurological, gastrointestinal diseases and anti-inflammatory agent [145,146,147,148,149,150,151,152,153,154,155,156].

Despite its high potency, this substance is not included in any of the United Nations Drug Conventions’ Schedules [3]. However, in Denmark, Latvia, Belgium, Lithuania, Sweden, Romania, Japan and Australia, these compounds are controlled. *S. divinorum* is considered an illegal drug in the United States of America, and its sale in Canada is also prohibited [3]. Other countries such as Germany, Poland, Croatia and Spain regulate its manufacture, and in Norway, Estonia and Finland this plant is legislated by the legislation of medicines [3]. Together with the consumption of *C. edulis* and *M. speciosa*, the consumption of *S. divinorum* is controlled by the United Nations Office on Drugs and Crime (UNODC) [3].

The consumption of this substance has expanded worldwide and, therefore, several analytical methodologies for the detection of salvinorin A have emerged (Table 8). Thus, several biological samples have been used for the development of these analytical methodologies, namely, urine [157,158,159,160], plasma [159,161], saliva [161], sweat [161], pericardial fluid [161], vitreous humor [161], blood [161] and hair [67]. Margalho et al. [161] quantified salvinorin A in pericardial fluid, vitreous humor, blood and plasma in the same analytical method. The quantity of the samples was reduced (100 µL-250 µL), being treated using the SPE technique [161]. Finally, the compounds were quantified using GC-MS equipment, and the method proved to be sensitive and selective, presenting LOD and LOQ of 5 ng/mg [161]. Moreno et al. [160] quantified salvinorin A in urine samples (200 µL) using MEPS as the sample pre-treatment technique. The samples were analyzed by GC-MS/MS equipment, with good recoveries (71%–80%) and good LOD and LOQ (5 ng/mL and 20 ng/mL, respectively) [160].

## 13. *Tabernanthe iboga* (Iboga)

**Figure 12:**
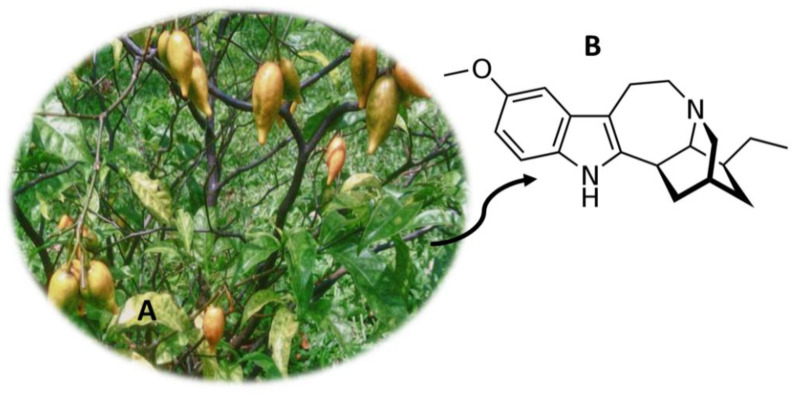
*Tabernanthe iboga* (**A**) and the main compound ibogaine (**B**).

*T. iboga* (Figure 12A) is a shrub from Central and West Africa [94,162]. This plant has been consumed for centuries in religious rituals of initiation into adulthood (Bwiti religion) in countries located in Central Africa and in the Congo basin [94,163]. The root barks of *T. iboga* contain psychoactive alkaloids, the majority of which are called ibogaine (Figure 12B) [162]. This compound, which consists of a monoterpene-indole alkaloid, is consumed orally in the form of hydrochloride, extracts of alkaloids or by consumption of the dry root bark [162,164,165]. When consuming, users experience stimulating and aphrodisiac properties, trance, energization and increased alertness [94,163]. The consumption of this substance also causes hallucinations that, in contrast to common hallucinogens, are more intense and realistic when experienced with closed eyes [162]. Despite the structure of ibogaine being similar to other hallucinogens, this compound has a different mode of action [162]. So far, its mechanism of action is not fully known, but it is known that it is able to act as an agonist of σ2 receptors and an antagonist of nicotinic α3β4 acetylcholine receptors and as an antagonist at N-methyl-d-aspartatetype (NMDA) glutamate receptors [164,165].

Throughout history, the extract of *T. iboga* has been used for other purposes, namely for fatigue and depression [166]. Currently, ibogaine is used in opioid detoxification [94,162]. Thus, this compound is legal in most countries, however, in Switzerland, Belgium, Australia, Sweden, France, Denmark and the United States of America, it is illegal [162]. Currently, there are several methodologies for quantifying *T. iboga* compounds, namely using biological samples such as plasma [167], urine [88,91], blood [89] and serum [88]. Pietsch et al. [88] developed an analytical method where they determined, among other compounds, ibogaine. For this, they used HPLC-PDA and HPLC-UV equipment, using 1 mL of serum and 1 mL of urine, which were pre-treated with SPE [88]. Furthermore, Björnstad et al. [91] developed an analytical method where they quantified ibogaine and other compounds. This analytical method had a LOD between 2 ng/mL and 10 ng/mL and presented a very easy sample preparation technique [91]. Only 50 µL of urine were used, which were diluted and injected directly into LC-MS/MS equipment [91].

## 14. Conclusions

Throughout this review, several plants/fungi that have psychoactive substances capable of inducing relaxing, stimulating or hallucinogenic effects were addressed. Toxicological aspects, some therapeutic properties and traditional uses were highlighted, as well as some of the analytical methods, developed in biological matrices, aimed at the detection of these substances.

Given the rapid emergence of these psychoactive substances in the abused drugs market, as well as the lack of legislation to control them, the development of new analytical methodologies is crucial. However, the lack of analytical standards to proceed with the development of chromatographic methods or the difficulty in finding plant specimens that allow scans of the compounds present in them constitute an enormous difficulty. Additionally, the fact that the compounds are usually present in very small amounts makes it even more difficult to develop and validate new methodologies, requiring the use of more sophisticated equipment, such as mass spectrometry detectors. Finally, the fact that the matrices of plant origin have several interferents also constitutes a difficulty, since it is necessary to apply a pre-treatment step to the sample. This procedure makes the development of methods more expensive and requires the use of organic solvents. In the future, the use of miniaturized extraction techniques should be prioritized in order to achieve the development of more economical methods that aim to use lower volumes of organic solvents and, consequently, be more environment-friendly.

## Figures and Tables

**Table 1:** Analytical methods for the determination of the main components of *Areca catechu*.

Compounds	Sample (Amount)	Sample Preparation	Analytical Technique	Limits of Detection	Limits of Quantitation	Recovery (%)	Reference
Arecoline	Teeth (50 mg)	Pulverization and sonication (methanol)	LC-MS/MS (ESI); LC-HR-ToF-MS (ESI)	-	-	-	[28]
Arecoline	Saliva (950 mL)	LLE (ethylacetate)	HPLC-UV-VIS	-	-	-	[29]
Arecoline	Hair (50 mg)	Pulverization; alkaline digestion (NaOH 12 M) and LLE (cloroform/isopropanol (95:5, *v/v*)	LC-MS (ESI)	0.09 μg/g	0.3 μg/g	81.2 ± 2.6	[32]
Arecoline	Meconium (1000 mg), urine (1 mL) and cord serum (1 mL)	LLE (chloroform/isopropanol (95:5, *v/v*))	LC-MS (ESI)	0.0004–0.001 μg/g	0.001–0.005 μg/g	86.5–90.7	[27]
Arecoline	Breast milk (1 mL)	LLE (chloroform/isopropanol (95:5, *v/v*))	LC-MS/MS (ESI)	16 μg/L	50 μg/L	76.8–84.7	[31]
Arecoline, Arecaidine and N-methylnipecotic acid	Saliva (0.05 mL)	PP (acetonitrile)	LC-MS/MS (ESI)	0.156 μg/L	1.25 μg/L	72.5–100.1	[30]

Caption: ESI (electrospray ionization); HPLC (high-performance liquid chromatography); HR (high resolution); LC (liquid chromatography); LLE (liquid-liquid extraction); MS (mass spectrometry); MS/MS (tandem mass spectrometry); PP (protein precipitation); ToF (time of flight); UV-VIS (ultraviolet-visible detector).

**Table 2:** Analytical methods for the determination of the main components of Ayahuasca.

Compounds	Sample (Amount)	Sample Preparation	Analytical Technique	Limits of Detection	Limits of Quantitation	Recovery (%)	Reference
5-OH-DMT, DMK, Harmol, Harmalol, NMT, 5-MeO-DMT, 2-MTHBC, THH, DMT-NO, Harmine and Harmaline	Urine (0.1 mL)	Dilution (90% water– 0.1% formic acid:10% acetonitrile–0.1% formic acid) and enzyme hydrolysis (glucuronidase–sulfatase–acetate buffer)	LC-MS/MS (ESI)	0.04–0.57 μg/L	5 μg/L	-	[65]
DMT, Harmine, Harmaline, THH, Harmol and Harmalol	Plasma (1 mL)	LLE (n-pentane) and SPE (C_18_)	GC–NPD and HPLC–FLD	-	0.3-1.6 μg/L	74–87	[63]
DMT, Harmine, Harmaline, THH, Harmol, Harmalol, 5-OH-DMT, THH-OH, DMK, NMT, 5-MeODMT, 2-MTHBC, DMT-NO	Blood (0.2 mL)	PP (96-well plates), Dilution (formic acid (0.1% in water); formic acid (0.1% inacetonitrile))	LC–MS/MS (HESI)	0.09–0.45 μg/L	1.0 μg/L	60.28–76.31	[62]
DMT, Harmine, Harmaline, THH	Plasma (1 mL)	SPE (C_18_)	LC–MS/MS (ESI)	0.1 μg/L	0.2–0.4 μg/L	88.4–107.7	[64]
DMT	Hair (25 mg)	Hydrolysis (M3 reagent)	UHPLC-MS/MS (ESI)	0.01–0.02 μg/g	0.03–0.05 μg/g	79.6–97.4	[67]
DMT and DMT-NO	Urine (0.1 mL)	Dilution (97:3 water with 0.1% formic acid:acetonitrile with 0.1% formic acid)	LC-MS/MS (ESI)	-	5.0 μg/L	-	[66]

Caption: ESI (electrospray ionization); FLD (fluorescence detector); GC (gas chromatography); HESI (heated electrospray); HPLC (high-performance liquid chromatography); LC (liquid chromatography); LLE (liquid-liquid extraction); MS/MS (tandem mass spectrometry); NPD (nitrogen–phosphorus detector); PP (protein precipitation); SPE (solid-phase extraction); UHPLC (ultrahigh-performance liquid chromatography).

**Table 3:** Analytical methods for the determination of the main components of *Catha edulis*.

Compounds	Sample (Amount)	Sample Preparation	Analytical Technique	Limits of Detection	Limits of Quantitation	Recovery (%)	Reference
Cathinone, Cathine and Phenylpropanolamine	Urine (0.2 mL)	SPE (C_8_)	GC-MS (EI)	<10µg/L	-	73–82	[72]
d-cathine (d-norpseudoehedrine), ephedrine, methcathinone, 1-(4-methoxyphenyl)-propan-2-amine, mephedrone, methedrone, 2,5-dimethoxy-4-methylamphetamine, 4-bromo-2,5-dimethoxyamphetamine, 2,5-dimethoxyphenethylamine, 4-bromo-2,5-dimethoxyphenethylamine, 4-iodo-2,5-dimethoxyphenethylamine, 2-[2,5-dimethoxy-4-(ethylthio)phenyl]ethanamine, 2,5-dimethoxy-4-isopropylthiophenethylamine and 2-[2,5-dimethoxy-4-(propylthio)phenyl]ethanamine	vitreous humor (0.1 mL), pericardial fluid (0.25 mL) and whole blood (0.25 mL)	SPE (Oasis^®^ MCX)	GC-MS (EI)	5 µg/L	5 µg/L	100	[74]
Cathinone, Methcathinone, Ethcathinone, Amfepramone, Mephedrone, Flephedrone, Methedrone, Methylone, Butylone, Cathine, Norephedrine, Ephedrine, Pseudoephedrine, Methylephedrine and Methylpseudoephedrine	Blood (0.3 mL)	PP (methanol) and Ultrafiltration	LC-MS/MS (ESI)	0.5–3 μg/L	-	87–106	[70]
cathinone, flephedrone, buphedrone, 4-MTA, α-PVP, methylone, 2C-P, ethylone, pentylone, MDPV and bromo-dragonFLY	whole blood (0.25 mL)	SPE (Oasis^®^ MCX)	GC-MS	40–5 µg/L	40–5 µg/L	70.3–116.6	[75]
Cathine, Cathinone, Methcathinone and Ephedrine	Oral fluid (0.5 mL)	LLE (ethyl acetate)	GC-MS (EI)	10.0 μg/L	20.0 μg/L	-	[73]

Caption: EI (electron impact); ESI (electrospray ionization); GC (gas chromatography); LC (liquid chromatography); LLE (liquid-liquid extraction); MS (mass spectrometry); MS/MS (tandem mass spectrometry); PP (protein precipitation); SPE (solid-phase extraction).

**Table 4:** Analytical methods for the determination of the main components of *Datura stramonium* and *Mandragora officinarum*.

Compounds	Sample (Amount)	Sample Preparation	Analytical Technique	Limits of Detection	Limits of Quantitation	Recovery (%)	Reference
Hyoscyamine and Scopolamine	Serum (0.5 mL) and Urine (0.5 mL)	SPE (Extrelut1)	GC-MS (EI)	5.0 μg/L	-	>80	[90]
Atropine, DMT, Ephedrine, Harmaline, Harmine, Ibogaine, LSA, Psilocin, Scopolamine and Yohimbine	Urine (0.05 mL)	Dilution (distilled water)	LC–MS/MS (ESI)	2.0–10.0 μg/L	-	-	[91]
α-lobeline, α-solanine, Aconitine, Ajmaline, Atropine, Brucine, Cephalomannine, Colchicine, Convallatoxin, Cymarine, Cytisine, Digitoxin, Digoxin, Emetine, Gelsemine, Ibogaine, Jervine, Kavain, Lanatoside C, Lupanine, Mitragynine, Neriifolin, Oleandrin, Ouabain, Paclitaxel, Physostigmine, Pilocarpine, Podophyllotoxin, Proscillaridin A, Reserpine, Retrorsine, Ricinine, Scopolamine, Senecionine, Sparteine, Strophanthidin, Strychnine, Veratridine and Yohimbine	Blood (1 mL)	SPE (HLB Oasis ®)	UHPLC-MS/MS (ESI)	0.1–1.6 μg/L	10 μg/L	33–106	[89]

Caption: EI (electron impact); ESI (electrospray ionization); GC (gas chromatography); LC (liquid chromatography); MS (mass spectrometry); MS/MS (tandem mass spectrometry); SPE (solid-phase extraction); UHPLC (ultrahigh-performance liquid chromatography).

**Table 5:** Analytical methods for the determination of the main components of *Mitragyna speciosa*.

Compounds	Sample (Amount)	Sample Preparation	Analytical Technique	Limits of Detection	Limits of Quantitation	Recovery (%)	Reference
Mitragynine, 7-hydroxymitragynine, Speciociliatine, Speciogynine and Paynantheine	Urine (1 mL)	SPE (PolyChrom ClinII 3 cm^3^ (35 mg))	LC-Q/TOF-MS	0.25–1 μg/L	0.5–1 μg/L	-	[122]
Mitragynine, 7-hydroxy-mitragynine, 5-desmethyl-mitragynine, 17-desmethyldihydromitragynine and mitraphylline	Urine (0.2 mL)	Hydrolysis (β-Glucuronidase) and LLE (methyl tert-butyl ether)	LC-MS/MS (ESI)	-	1 μg/L	-	[123]
Mitragynine, 16-carboxy mitragynine and 9-O-demethyl mitragynine	Urine (1 mL)	Hydrolysis (β-glucuronidase/arylsulfatase) and SPE (Bond Elut Certify (200 mg, 3 mL) and Abs Elut-Nexus SPE (60 mg, 3 mL))	LC-MS/MS (ESI)	-	1–50 μg/L	-	[121]
Mitragynine and 7-hydroxymitragynine	Urine (1 mL)	Dilution (water with 0.1% formic acid)	LC-MS/MS (ESI)	0.012–0.069 μg/L	0.0356–0.215 μg/L	-	[124]

Caption: ESI (electrospray ionization); LC (liquid chromatography); LLE (liquid-liquid extraction); MS (mass spectrometry); MS/MS (tandem mass spectrometry); SPE (solid-phase extraction); Q/ToF (time of flight).

**Table 6:** Analytical methods for the determination of the main components of *Piper methysticum*.

Compounds	Sample (Amount)	Sample Preparation	Analytical Technique	Limits of Detection	Limits of Quantitation	Recovery (%)	Reference
Kavain, Dihydrokavain, Methysticin, Dihydromethysticin and Desmethoxyyangonin	Urine (0.1 mL) and Plasma (0.1 mL)	PP (Methanol), LLE (ethyl acetate) and SPE (SOLA HRP cartridge)	UHPLC-MS/MS (HESI)	0.015–0.137 μg/L	0.0457–0.4165 μg/L	-	[129]
Kavain	Hair (29–50 mg)	Decontamination (methylene chloride) and digestion (methanol)	GC-MS/MS (EI)	0.030 μg/g	0.1 μg/g	-	[127]
Kavain, *p*-hydroxykavain, *p*-hydroxy-5,6-dehydrokavain and *p*-hydroxy-7,8-dihydrokavain	Blood (1 mL), Urine (1 mL) and Serum (1 mL)	LLE (dichlormethane:diethylether (7:3, *v*/*v*))	HPLC-DAD and LC-MS	1 μg/L	5 μg/L	91–97	[130]
Kavain, 7,8-dihydrokavain, Yangonin, 5,6-dehydrokavain, 12-hydroxy-5,6-dehydrokavain, Methysticin, 7,8-dihydromethysticin, 11-hydroxy-5,6-dehydrokavain, 12-hydroxykavain and 12-hydroxy-7,8-dihydrokavain	Hair (21–253 mg)	Decontamination (HPLC water, acetone and petroleumbenzene) and digestion (methanol)	HPLC-DAD, LC-MS/MS (ESI) e GC/TOF-MS	-	-	-	[128]

Caption: DAD (diode array detector); EI (electron impact); ESI (electrospray ionization); GC (gas chromatography); HESI (heated electrospray); HPLC (high performance liquid chromatography); LC (liquid chromatography); LLE (liquid-liquid extraction); MS (mass spectrometry); MS/MS (tandem mass spectrometry); PP (protein precipitation); SPE (solid-phase extraction); ToF (time of flight); UHPLC (ultrahigh-performance liquid chromatography).

**Table 7:** Analytical methods for the determination of the main components of the *Psilocybe* genus.

Compounds	Sample (Amount)	Sample Preparation	Analytical Technique	Limits of Detection	Limits of Quantitation	Recovery (%)	Reference
Psilocin glucuronide and Psilocin	Urine (0.1mL)	Enzymatic hydrolyses (β-glucuronidase), alkaline hydrolyses (potassium hydroxide) acid hydrolysis (concentratedhydrochloric acid) and deproteinization (methanol)	LC-MS (ESI) and LC-MS/MS (ESI)	0.5 μg/L	-	-	[138]
Psilocin glucuronide	Serum (0.1mL)	Enzymatic hydrolysis (β-glucuronidase) and deproteinization (methanol)	LC-MS (ESI) and LC-MS/MS (ESI)	0.5 μg/L	-	-	[139]
Mescaline, DMT, Psilocin, Psilocybin and Salvinorin A	Hair (25 mg)	Hydrolysis (M3reagent)	UHPLC-MS/MS (ESI)	0.01–0.02 μg/g	0.03–0.05 μg/g	79.6–97.4	[67]

Caption: ESI (electrospray ionization); LC (liquid chromatography); MS (mass spectrometry); MS/MS (tandem mass spectrometry); UHPLC (ultrahigh-performance liquid chromatography).

**Table 8:** Analytical methods for the determination of the main components of *Salvia divinorum*.

Compounds	Sample (Amount)	Sample Preparation	Analytical Technique	Limits of Detection	Limits of Quantitation	Recovery (%)	Reference
Salvinorin A	Urine (20 mL)	LLE (chloroform) and SPME (85 µm polyacrylate fiber)	GC × GC − ToF-MS	4–200 μg/L	-	-	[157]
Salvinorin A	Urine (1 mL)	SPE (Waters Oasis ^®^HLB)	LC-MS (ESI)	5 μg/L	2.5 μg/L	-	[158]
Salvinorin A	Plasma (1 mL), Urine (1 mL), Saliva (1 mL) and Sweat (1 patch cut into littlepieces)	LLE (chloroform/isopropanol (9:1, *v*/*v*))	GC-MS (EI)	3–5 μg/L	10–15 μg/L	77.1–92.7	[159]
Salvinorin A	Pericardial fluid (0.25 mL), Vitreous humor (0.1 mL), Blood (0.25 mL) and Plasma (0.25 mL)	SPE (Waters Oasis^®^HLB)	GC-MS (EI)	5.0 μg/L	5.0 μg/L	-	[161]
Salvinorin A	Urine (0.2 mL)	MEPS (C_18_)	GC-MS/MS (EI)	5.0 μg/L	20 μg/L	71–80	[160]

Caption: EI (electron impact); ESI (electrospray ionization); GC (gas chromatography); GC × GC (bidimensional gas chromatography); LC (liquid chromatography); LLE (liquid-liquid extraction); MEPS (microextraction by packed sorbent); MS (mass spectrometry); MS/MS (tandem mass spectrometry); SPE (solid-phase extraction); ToF (time of flight).
